# A survey in rural China of parent-absence through migrant working: the impact on their children's self-concept and loneliness

**DOI:** 10.1186/1471-2458-10-32

**Published:** 2010-01-23

**Authors:** Li-Juan Liu, Xun Sun, Chun-Li Zhang, Yue Wang, Qiang Guo

**Affiliations:** 1 Office of Medical Education, Training Department, Second Military Medical University, Shanghai, PR China; 2 Changzheng Hospital, Second Military Medical University, Shanghai, PR China; 3 Training Department, Second Military Medical University, Shanghai, PR China

## Abstract

**Background:**

Following the rapid increase of migrant workers in China, the number of "absent migrant parents" children is also rising fast. The "absent migrant parents" children might have an insecure relationship with their parents, have a different view of them, and be prone to have the feeling of loneliness. The purpose of the study was to compare the self-concept and loneliness between the "absent migrant parents" children and comparison children, to examine the relationship between self-concept and loneliness among the two groups, and to study the predictors of self-concept among the two groups.

**Methods:**

Participants were 230 "absent migrant parents" children and 250 comparison children in the rural area of a county, China. The self-concept and loneliness of children were assessed using Piers-Harris Self-Concept Scale and Childhood Loneliness Scale.

**Results:**

The "absent migrant parents" children were more likely to dislike their parents or be uncertain whether they like their parents, and they reported less time spent in physical and leisure time activities, higher loneliness and lower self-concept in comparison with the comparison children. Loneliness was significantly negatively correlated with all the dimensions of self-concept among the two groups. Regression analysis showed that self-concept was positively related to the relationship with parents and guardians and time spent in physical and leisure activities among the "absent migrant parents" children. The same factors (except the relationship with guardians) were found for self-concept among the comparison children.

**Conclusions:**

The "absent migrant parents" children were more inclined to have lower self-concept and higher loneliness. The lower self-concept seemed to contribute to the higher loneliness of the "absent migrant parents" children. The lower self-concept of the "absent migrant parents" children was mainly related with their relationship with parents and guardians. The acceptance and support from their parents could not be fully replaced by that from their guardians.

## Background

Following the rapid increase of migrant workers in China [1-3], the number of "absent migrant parents" children who grow up outside the parental environment or with limited parental involvement because their parents are working far away from home is also rising fast. Statistics show that by 2004, this group had reached 22 million.

The relationship between parent and child is an important feature for the development of a child [4-6]. In the first month after birth, a child seeks proximity to the parent (mother), and if the parent responds to the needs of the child, the child will create a secure attachment with the parent, from which it will explore the world [7].

Self-concept is the cognitive or thinking aspect of self (related to one's self-image and self-esteem) and generally refers to "the totality of a complex, organized, and dynamic system of learned beliefs, attitudes and opinions that each person holds to be true about his or her personal existence". In the Attachment Theory, John Bowlby and Mary Ainsworth claim that during the early years, while the child acquires the capacity for self-regulation, the mother is a child's ego and superego [8,9]: It is not surprising that during infancy and early childhood the functions related to self-regulation are either not operating at all or are operating poorly. During this phase of life, the child is therefore dependent on his or her mother performing these functions for him or her. She orients him or her in space and time, provides his or her environment, permits the satisfaction of some impulses, and restricts others. Gradually the child learns these arts himself or herself, and as he or she does, the skilled parent transfers the roles to him or her, and he or she gradually developed his or her attitude and assessment toward his or her own self. Parental acceptance and support is important for children's self-concept development [10]. The basis of self-concept is that an affectionate parent positively evaluates his or her child and provides emotional support to the child. A study of 1668 secondary school students showed that better relation with parents was associated with higher general, academic, social and physical ability of self-concepts [11].

Self-concept is considered an important psychological attribute and is thought to be associated with mental health. Numerous empirical studies have demonstrated the importance of self-concept for children's psychological growth [12,13]. Low self-concept is seen in various psychiatric disorders. A previous study showed that there was a strong relationship between self-concept and loneliness [12].

Growing up outside the parental environment, the "absent migrant parents" children could not discuss their thoughts, their situation or their problems with their parents. They might have an insecure relationship with their parents, have a lower attitude and assessment toward themselves, and be prone to have the feeling of loneliness. However, few studies have focused on the self-concept and loneliness of the "absent migrant parents" children or studied the predictors of self-concept among the "absent migrant parents" children. This study was carried out with three main objectives: to compare the self-concept and loneliness between the "absent migrant parents" and comparison children, to examine the relationship between self-concept and loneliness among the two groups, and to study the predictors of self-concept among the two groups.

## Methods

### Location

Yuan'an is an inland mountainous county in Hubei province. In 2004, the population of Yuan'an is 206,801. The geographic area of the town is 1752.3 km^2^, which includes 7 small towns and 110 villages, as measured in 2005.

### Subjects

Multistage stratified random cluster sampling was used. First, the 7 small towns in Yuan'an County were stratified as three groups according to the developmental level of economy, and one small town was randomly selected from every group. Second, the schools in the rural area of every selected small town were stratified as three groups according to the level of education, and one school was randomly selected from every group. In all, nine schools were randomly selected.

In the selected nine schools, the "absent migrant parents" children from grade 4 to 6 were identified by asking whether they grew up outside the parental environment or with limited parental involvement because both of their parents were working far away from home. Using a case-by-case matching procedure, the comparison children were randomly selected from the same grade. A standardized questionnaire was sent to the "absent migrant parents" children and comparison children selected.

In 2008, a total of 230 "absent migrant parents" children and 250 comparison children in the rural areas of Yuan'an County were invited to participate in the study. The informed consent was obtained both from the children and their parents or guardians. This study was approved by the Biological and Medical Ethics Committee, Second Military Medical University. There were twenty four children in all returned incomplete data, and only 216 "absent migrant parents" children and 240 comparison children completed the questionnaire thoroughly. The response rate of effective questionnaires was 95.0% (456/480).

### Instruments

The 80-item Piers-Harris Self-Concept Scale is a quantitative self-report measure of children's self-concepts. The scale includes six dimensions: behavior, intellectual and school status, physical appearance and attributes, anxiety, popularity, and happiness and satisfaction [14]. The total number of items is 80, of which 40 items are positive and the other 40 items negative. All the items require the subjects to answer "yes" or "no" only. Examples of the items are as follows: I am clever; I am lucky; I am always sad; I am always happy; and I feel shy. Score "0" is assigned to each positive item but with answer stated as "no", and to each negative item but with answer stated as "yes". The total scores for the scale range from 0 to 80, with higher scores indicating better self-concept. The total scores range from 0 to 16 for the behavior subscale, from 0 to 17 for the intellectual and school status subscale, from 0 to 13 for the physical appearance and attributes subscale, from 0 to 14 for the anxiety subscale, from 0 to 12 for the popularity subscale and from 0 to 10 for the happiness and satisfaction subscale. The Piers-Harris Self-Concept Scale has been used widely, with median test-retest reliability of 0.73 and estimates of internal consistency ranging from 0.88 to 0.93 [15]. The mean ± standard deviations of the total scale and the subscales for the comparison children aged from 8 to 16 years old in China were total Piers-Harris Self-Concept Scale = 51.66 ± 11.47, behavior subscale = 11.97 ± 2.97, intellectual and school status = 8.89 ± 3.27, physical appearance and attributes subscale = 6.40 ± 2.81, anxiety subscale = 9.35 ± 2.85, popularity subscale = 8.49 ± 2.20, and happiness and satisfaction subscale = 7.12 ± 1.98 [16].

The Childhood Loneliness Scale is a 24-item self-report measure that comprised 16 primary items, which were designed to tap children's feelings of loneliness in the school context [17]. A sample item is "I get along with my classmates" (reverse-scored). The eight additional items are filler items that concentrated on children's hobbies and preferred activities and school subjects, for example, "I watch TV a lot." All items are answered on a 5-point scale ranging from 1 (not at all) to 5 (always). Participants' scores on the Childhood Loneliness Scale, therefore, could range between 16 and 80, with higher scores indicating higher degrees of loneliness. To avoid biasing the children's responses, the instrument was presented to them as the "Children's Interests Scale". In terms of internal consistency, the Children's Loneliness Scale was one of the most reliable self-rating measures of loneliness for children. The Cronbach alpha of the scale reaches 0.80 or above [18]. The mean ± standard deviation of the scale for the comparison children from grade 5 to 6 in the rural area of China was 25.97 ± 6.83 [19].

Relationship between the "absent migrant parents" children and their guardians was examined by asking "Do you like your guardians? (strongly dislike, moderately dislike, uncertain, moderately like, strongly like). Relationship between the children and their parents was also examined by asking "Do you like your parents? (strongly dislike, moderately dislike, uncertain, moderately like, strongly like).

Information on subject age, gender (male, female), grade (four, five, six), family economic status (very bad, bad, middle, well, very well), time spent in leisure time activities (very little, little, moderate, much, very much), such as reading, writing and handicraft, etc., and time spent in physical activities (very little, little, moderate, much, very much), such as running, playing basketball and swimming were collected from subject self-report.

Answers to questions such as who cared for the subject, how long they were separated from their parents, how often their parents returned (once every month, once every 2-6 months, once every 7-12 months, and once every more than a year), whether their parents communicated with them by telephone frequently (yes, no), were collected from the "absent migrant parents" subject self-report.

### Data analysis

Student's t-tests were used to compare the age, loneliness and self-concept between the "absent migrant parents" group and comparison group. Chi-Square tests were used to compare the gender, grade, family economic status, relationship with parents, time spent in leisure time activities and time spent in physical activities between the two groups. Chi-Square tests were used to examine the relationships between the "absent migrant parents" children's relationship with their parents and the parental contact including how often the parents returned home and whether they communicated with their children by telephone frequently. The association between self-concept and loneliness in the two groups were explored with Pearson's correlations. To identify factors predicting self-concept among the "absent migrant parents" children and comparison children, multiple linear regression models with conditional stepwise analysis were used. One-way ANOVA was used to examine the relationship between self-concept and the frequency that the parents returned home among the "absent migrant parents" group. Student's t-test was used to examine the relationship between self-concept and the frequency that parents communicated with the children among the "absent migrant parents" group. P < 0.05 was considered significant. All data were analyzed with the SPSS 10.0 statistical analysis software package [20].

## Results

### Demographic and economic characteristics

Ages of these children ranged from 8 to 15 years (mean = 10.95 ± 1.11 years) for the "absent migrant parents" group and from 8 to 14 years (mean = 11.05 ± .99 years) for the comparison group. The majority of the "absent migrant parents" children (88.9%) and comparison children (88.3%) were between 10 and 12 years. Student's t-test showed that there was no significant difference between the two groups in age (p = .307). Chi-Square tests showed that there were no significant differences between the "absent migrant parents" group and comparison group in gender (male: 44.4% vs. 47.5%; female: 55.6% vs. 52.5%; p = .572), grade (four: 33.3% vs. 32.5%; five: 35.2% vs. 34.2%; six: 31.5% vs. 33.3%; p = .915) and family economic status (very bad: 1.9% vs. 1.3%; bad: 4.6% vs. 3.3%; middle: 65.7% vs. 68.8%; well: 26.9% vs. 25.4%; very well: 0.9% vs. 1.3%; p = .894).

### Relationships with parents and guardians

Time of the "absent migrant parents" children outside the parental environment ranged from 1 to 12 years (mean = 3.85 ± 3.11 years). The ages of these children when they were separated with their parents ranged from 1 to 13 years (mean = 7.34 ± 3.22 years). More than half (62.0%) of their parents returned home once every 2-12 months, and the others returned home once every month. Most (85.6%) of their parents communicated with them by telephone frequently, and the others (14.4%) didn't communicate with them frequently.

Almost all of the "absent migrant parents" children (97.2%) were cared for by their grandfathers and grandmothers, and the others (2.8%) were cared for by their other relatives. When the "absent migrant parents" children were asked whether they like their guardians, most of them reported that they liked their guardians (Strongly dislike: 0.0%; Moderately dislike: 3.7%; Uncertain: 19.4%; Moderately like: 69.4%; Strongly like: 7.5%). When all the children were asked whether they like their parents, the results showed that the "absent migrant parents" children were more prone to be uncertain or dislike their parents in comparison with the comparison children (Moderately dislike: 3.7% vs. 1.6%; Uncertain: 64.8% vs. 9.6%; Moderately like: 18.5% vs. 29.2%; Strongly like: 13.0% vs. 59.6%; p < .001). The results also showed that the more frequently the parents returned home and communicated with their children, the better relationship they would get with their children (Table 1 and Table 2).

**Table 1:** Distributions of the "absent migrant parents" children^a^'s relationship with parents regarding how often the parents returned home

*How often the parents returned home*	*Relationship with parents (n/percent)*				*Total (n/percent)*	*P value*
			
	*Moderately dislike*	*Uncertain*	*Moderately like*	*Strongly like*		
Once every month	0/0.0	38/46.3	20/24.4	24/29.3	82/100.0	< .001
Once every 2-6 months	0/0.0	47/70.1	16/23.9	4/6.0	67/100.0	
Once every 7-12 months	8/11.9	55/82.1	4/6.0	0/0.0	67/100.0	

NOTE: ^a^N = 216

**Table 2:** Distributions of the "absent migrant parents" children^a^'s relationship with their parents regarding their communication

*Whether parents communicated with them frequently*	*Relationship with parents (n/percent)*				*Total (n/percent)*	*P value*
			
	*Moderately dislike*	*Uncertain*	*Moderately like*	*Strongly like*		
Yes	1/0.5	116/62.7	40/21.6	28/15.1	185/100.0	< .001
No	7/22.6	24/77.4	0/0.0	0/0.0	31/100.0	

NOTE: ^a^N = 216

### Time spent in activities

Chi-Square tests showed that the "absent migrant parents" children were more likely to spend less time in both physical (Little: 40.7% vs. 26.7%; Moderate: 44.4% vs. 57.0%; Much: 14.8% vs. 16.3%; p = .005) and leisure time (Little: 14.8% vs. 9.6%; Moderate: 48.1% vs. 40.0%; Much: 37.0% vs. 50.4%; p = .012) activities in comparison with the comparison children.

### Self-concept and loneliness

The total Piers-Harris Self-Concept Scale score and almost all the subscale scores including behavior, intellectual and school status, anxiety, popularity, and happiness and satisfaction among the "absent migrant parents" children were significantly lower than that among the comparison children (Table 3).

**Table 3:** The differences of loneliness and self-concept between the "absent migrant parents"^a ^children and comparison^b ^children

*Factors*	*"Absent migrant parents" group (mean ± SD score)*	*Comparison group (mean ± SD score)*	*P value*
**Childhood Loneliness Scale^c^**	29.85 ± 7.54	27.98 ± 8.52	.001
**Piers-Harris Self-Concept Scale**			
Behavior^d^	10.73 ± 2.77	11.98 ± 2.70	< .001
Intellectual and school status^e^	8.25 ± 3.09	8.97 ± 3.44	.001
Physical appearance and attributes^f^	6.41 ± 2.56	6.47 ± 2.97	.745
Anxiety^g^	8.64 ± 3.25	9.33 ± 2.92	.003
Popularity^h^	8.19 ± 1.30	8.51 ± 1.83	.001
Happiness and satisfaction^i^	6.69 ± 1.89	7.17 ± 2.19	.002
Total score^j^	47.91 ± 11.97	51.42 ± 12.62	.002

NOTE: ^a^N = 216; ^b^N = 240; ^c^Score ranged from 16 to 80, with higher scores indicating higher degrees of loneliness; ^d^Score ranged from 0 to 16, with higher scores indicating fewer problems in behavior; ^e ^Score ranged from 0 to 17, with higher scores indicating fewer problems in intellectual and school status; ^f^Score ranged from 0 to 13, with higher scores indicating fewer problems in physical appearance and attributes; ^g^Score ranged from 0 to 14, with higher scores indicating fewer problems in anxiety; ^h^Score ranged from 0 to 12, with higher scores indicating fewer problems in popularity; ^i^Score ranged from 0 to 10, with higher scores indicating fewer problems in happiness and satisfaction; ^j^Score ranged from 0 to 80, with higher scores indicating better self-concept

In our study, the "absent migrant parents" children had higher loneliness mean score as measured by the Childhood Loneliness Scale compared with the comparison children (Table 3). Correlational analyses indicated that loneliness as measured by the Childhood Loneliness Scale was negatively associated with self-concept as assessed by the Piers-Harris Self-Concept Scale in both the "absent migrant parents" and comparison children. In fact, loneliness was significantly negatively correlated with all the dimensions of self-concept including behavior, intellectual and school status, physical appearance, anxiety, popularity, and happiness and satisfaction in the two groups (Table 4).

**Table 4:** Correlations of self-concept with loneliness between the "absent migrant parents"^a ^children and comparison^b ^children

*Peer's-Harris Children's-concept Scale*	*Loneliness ("absent migrant parents" group)*		*Loneliness (comparison group)*	
	
	Pearson Correlation	*P *value	Pearson Correlation	*P *value
Behavior	-.482	< .001	-.349	< .001
Intellectual and school status	-.628	< .001	-.483	< .001
Physical appearance	-.523	< .001	-.355	< .001
Anxiety	-.494	< .001	-.432	< .001
Popularity	-.606	< .001	-.566	< .001
Happiness and satisfaction	-.481	< .001	-.413	< .001
Total score	-.684	< .001	-.526	< .001

NOTE: ^a^N = 216; ^b^N = 240

### Predictors of self-concept

To identify the predictors of self-concept among the "absent migrant parents" children and comparison children, linear regression models with conditional stepwise analysis were carried out using self-concept as the dependent variable and gender, age, relationship with guardians, relationship with parents, time spent in leisure time activities, and time spent in physical activities as independent variables (Table 5).

**Table 5:** Factors predicting the self-concept among the "absent migrant parents"^a ^children and comparison^b ^children. Results of multivariate, linear analysis

*Factor*	*"Absent migrant parents" group*		*Comparison group*	
	
	*B*	*Beta*	*B*	*Beta*
Relationship with guardians^c^	9.442 ***	.491	-	-
Relationship with parents^d^	5.936 ***	.380	9.565 ***	.497
Time spent in physical activities^e^	3.553 ***	.210	5.443 ***	.285
Time spent in leisure time activities^f^	1.491 ***	.086	3.457 *	.137
Gender^g^	-	.004		.003
Age	-	.035		.056

NOTE: ^a^N = 216; ^b^N = 240; B = Unstandardized Coefficients; Beta = Standardized Coefficients; ^c^Strongly dislike = 1, moderately dislike = 2, uncertain = 3, moderately like = 4, strongly like = 5; ^d^Strongly dislike = 1, moderately dislike = 2, uncertain = 3, moderately like = 4, strongly like = 5; ^e^Very little = 1, little = 2, moderate = 3, much = 4, very much = 5; ^f^Very little = 1, little = 2, moderate = 3, much = 4, very much = 5; ^g^Male = 1, female = 2; ***p < 0.001; **p < 0.01; *p < 0.05

In Table 5, the predictors of self-concept among the "absent migrant parents" children and comparison children were shown. Except the relationships with guardians, the same factors were found for self-concept between the two groups. Relationship with guardians was the strongest predictor of self-concept among the "absent migrant parents" children. Better relationship with the grandparents or other guardians was positively related with better self-concept among the "absent migrant parents" children. Positive relationships were found between self-concept and relationship with parents among the "absent migrant parents" children and comparison children. Relationship with parents was the second strongest predictor of self-concept among the "absent migrant parents" children and the strongest predictor among the comparison children.

Our results also showed that self-concept had a "dose" effect with the level of parental contact among the "absent migrant parents" children, with the lower contact having lower self-concept (Table 6).

**Table 6:** The "absent migrant parents" children^a^'s self-concept between different levels of parental contact

*Parental contact factors*	*Mean*	*SD*	*P value*
**How often the parents returned home**			
once every one month	53.98	10.80	< .001
once every 2-6 months	51.20	6.81	
once every 7-12 months	37.18	10.18	
**Whether parents communicated with them frequently**			
Yes	50.27	10.66	< .001
No	33.78	9.48	

NOTE: ^a^N = 216

There were also positive relationships between self-concept and time spent in physical and leisure time activities among the "absent migrant parents" children and comparison children (Table 5).

## Discussion

The study was a cross-sectional study, which was limited by the fact that it was carried out at one time point and given no indication of the sequence of events-whether exposure occurred before, after or during the onset of the outcome. This being so, it was difficult to infer the causality.

Parental acceptance and support encourage the child to explore personal limits and discover competencies, which is important for self-concept development [10]. The level of self-concept in the comparison group of our study was similar with that of previous studies in China [16]. The "absent migrant parents" children grow up outside the parental environment or with limited parental involvement because their parents are working far away from home. The socioeconomic status did not significantly differ between the "absent migrant parents" group and comparison group. There was comparability between the two groups. Our study showed that the "absent migrant parents" children demonstrated significantly lower self-concept compared with the comparison children. In fact, almost all the dimensions of self-concept including behavior, intellectual and school status, anxiety, popularity, and happiness and satisfaction among the "absent migrant parents" children were lower than that among the comparison children.

Self-concept is considered an important psychological attribute and thought to be associated with mental health. Low self-concept is seen in various psychiatric disorders. Numerous empirical studies have demonstrated the importance of self-concept for children's psychological growth [12,13]. Our study also demonstrated there were negative relationships between loneliness and all the dimensions of self-concept among the "absent migrant parents" and comparison children. Although these effects were not unique for the "absent migrant parents" children, they were quite important, because the "absent migrant parents" children were more inclined to have lower self-concept. To decrease the loneliness of the "absent migrant parents" children, it might be helpful to increase their self-concept.

To increase the self-concept of the "absent migrant parents" children, factors predicting their self-concept must be found. Self-concept is not innate, but is developed or constructed by the individual through interaction with the environment and reflecting on that interaction. Numerous empirical studies have demonstrated the importance of warm and accepting parenting for children's self-concept development [10,11]. The "absent migrant parents" children grow up outside the parental environment, and they spent much of their time with the guardians. In our study, relationship with guardians was the strongest predictor of self-concept, and relationship with parents was the second strongest predictor of self-concept among the "absent migrant parents" children. It was concluded that the quality of relationship with the guardians was important for the "absent migrant parents" children's self-concept development, which was consist with the attachment theory that in the presence of close alternate care the child could had better self-concept. The quality of relationship with parents was also important for the "absent migrant parents" children and comparison children's self-concept development. For the "absent migrant parents" children, the acceptance and support from the parents could not be fully replaced by that from the guardians. The self-concept had a "dose" effect with the level of parental contact among the "absent migrant parents" children, with the lower contact having lower self-concept, which suggested that there was a positive relationship between self-concept and the level of parental contact.

The relationship between parent and child was an important feature for the development of a child. It was the basis of a child's self-concept that a parent was affectionate, positively evaluates his or her child and provides emotional support to the child. However, the "absent migrant parents" children had poorer relationships with their parents in comparison with the comparison children, which partly explained the lower self-concept of the "absent migrant parents" children. To improve the relationship between the "absent migrant parents" children and their parents and increase the "absent migrant parents" children's self-concept, it would be helpful that the parents returned home and communicated with their children by telephone more frequently.

The Attachment Theory places special importance on the primary carer who is usually the birth mother. Therefore it would be useful to know if the relationship with parents was different for mothers and fathers, and whether mothers or fathers absence had different impact on the children's self-concept (For example, liking, communication etc). The absence of this information in our study limits both the theoretical interpretation as well as the interventions that could be planned (for example encouraging mothers in particular to remain with their children).

A Previous study has reported that increased level of sports participation had a positive impact on self-concepts [21], which was consistent with our study. Our study showed that time spent in physical and leisure time activities were both positively associated with self-concept among the "absent migrant parents" and comparison children. However, the "absent migrant parents" children spent less time in both physical and leisure time activities in comparison with the comparison children, which might partly explained the lower self-concept of the "absent migrant parents" children.

Childhood is a critical period for the development of self-concept. The public should pay more attentions and take serious actions to increase the self-concept of the "absent migrant parents" children. To ensure the children whose parents are working far away from home to grow up inside the parental environment, the Chinese government is using legislative means to ensure that migrant workers can choose to have their children by their side. In one of the meetings of China's legislative organizations, the Law Committee of the National People's Congress proposed a "draft of compulsory educational act" that requires local governments to give the children of migrant workers equal educational opportunities. This would enable more migrant workers to bring their children to live by their side. For those migrant workers who wouldn't have their children by their side, they should return home or communicate with their children by phone more frequently. At the same time, primary schools should change their current teaching methods to pay more attention to the cultivation of hobbies among children, and give children more time to pursuit their interests.

## Conclusions

The "absent migrant parents" children were more inclined to have lower self-concept and higher loneliness. There were negative relationships between self-concept and loneliness among the "absent migrant parents" and comparison children. The lower self-concept of the "absent migrant parents" children was mainly related with their worse relationship with parents and less time spent in physical and leisure time activities. The acceptance and support from their parents could not be fully replaced by that from their guardians. A potential weakness in the present study is that the study does not get a picture of the experiences of the"absent migrant parents" children. It would be interesting to investigate the reason for the children's dislike of separated parents. It could be the parents' leaving, or it could be that the relationship was poor before the leaving and the poor relationship created fewer bonds for parents to stay in the household.

## Competing interests

The authors declare that they have no competing interests.

## Authors' contributions

QG designed the study. LJL, XS, CLZ and QG conducted the study. LJL wrote the article under the assistance of QG. YW was the main coordinator of the study. All authors provided comments on the drafts and have read and approved the final version.

## Pre-publication history

The pre-publication history for this paper can be accessed here:

http://www.biomedcentral.com/1471-2458/10/32/prepub
